# Nanoneedle‐Based Electroporation for Efficient Manufacturing of Human Primary Chimeric Antigen Receptor Regulatory T‐Cells

**DOI:** 10.1002/advs.202416066

**Published:** 2025-04-15

**Authors:** Ningjia Sun, Cong Wang, William Edwards, Yikai Wang, Xiangrong L. Lu, Chenlei Gu, Samuel McLennan, Panicos Shangaris, Peng Qi, Daniela Mastronicola, Cristiano Scottà, Giovanna Lombardi, Ciro Chiappini

**Affiliations:** ^1^ Centre for Craniofacial and Regenerative Biology King's College London London SE1 9RT UK; ^2^ London Centre for Nanotechnology King's College London London WC2R 2LS UK; ^3^ Peter Gorer Department of Immunobiology School of Immunology & Microbial Sciences Faculty of Life Sciences & Medicine King's College London London SE1 7EH UK; ^4^ Department of Biosciences Centre for Inflammation Research and Translational Medicine Brunel University London London UB8 3PH UK; ^5^ Wenzhou Eye Valley Innovation Center Eye Hospital Wenzhou Medical University Zhejiang 325035 China; ^6^ School of Life Course & Population Sciences 10th Floor North Wing St Thomas’ Hospital King's College London London SE1 7EH UK; ^7^ Harris Birthright Research Centre for Fetal Medicine King's College London London SE1 7EH UK; ^8^ Department of Bioengineering Imperial College London London SW7 2AZ UK

**Keywords:** CAR‐T, electroporation, nanoneedle, nanoscale electroporation, non‐viral transfection, regulatory T cells, tregs

## Abstract

Regulatory T cells (Tregs) play a crucial role in moderating immune responses offering promising therapeutic options for autoimmune diseases and allograft rejection. Genetically engineering Tregs with chimeric antigen receptors (CARs) enhances their targeting specificity and efficacy. With non‐viral transfection methods suffering from low efficiency and reduced cell viability, viral transduction is currently the only viable approach for GMP‐compliant CAR‐Treg production. However, viral transduction raises concerns over immunogenicity, insertional mutagenesis risk, and high costs, which limit clinical scalability. This study introduces a scalable nanoneedle electroporation (nN‐EP) platform for GMP‐compatible transfection of HLA‐A2‐specific CAR plasmids into primary human Tregs. The nN‐EP system achieves 43% transfection efficiency, outperforming viral transduction at multiplicity of infection 1 by twofold. Importantly, nN‐EP preserves Treg viability, phenotype and proliferative capacity. HLA‐A2‐specific CAR‐Tregs generated using nN‐EP show specific activation and superior suppressive function compared to polyclonal or virally transduced Tregs in the presence of HLA‐A2 expressing antigen presenting cells. These findings underscore the potential of nN‐EP as a GMP‐suitable method for CAR‐Treg production, enabling broader clinical application in immune therapies.

## Graphical abstract

Low reproducibility and inefficient gene transfer hamper clinical manufacturing of chimeric antigen receptor regulatory T cells (CAR Tregs). Nanoneedle mediated electroporation generates primary human CAR Tregs under good manufacturing practice conditions with superior efficiency compared to viral transduction. The resulting CAR Tregs maintain their proliferative capacity, phenotype and acquire the expected targeted ability to suppress the proliferation of effector T cells.

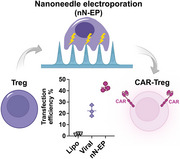

## 1 Introduction

T‐cell immunotherapies utilizing chimeric antigen receptors (CARs) have achieved remarkable success, particularly since 2017, following the FDA approval of anti‐CD19 CAR T‐cell therapy for B cell lymphoma.^[^ ^1^ ^]^ CARs are engineered fusion proteins that redirect T‐cells to target cells expressing specific antigens^[^ ^2^ ^]^ bypassing the need for antigen presentation.^[^ ^3^ ^]^ Regulatory T cells (Tregs) have garnered significant attention in clinical immunology for their ability to actively suppress excessive immune responses while maintaining immune homeostasis.^[^ ^4^, ^5^, ^6^, ^7^, ^8^, ^9^, ^10^ ^]^ This ability provides transformative advantages in the treatment of autoimmune diseases such as Type 1 Diabetes (T1D), inflammatory conditions like inflammatory bowel disease (IBD), and for improving the success of organ transplantation^[^ ^11^, ^12^, ^13^, ^14^, ^15^, ^16^, ^17^, ^18^, ^19^, ^20^, ^21^, ^22^, ^23^ ^]^ where CAR‐Tregs exhibit greater specificity and functionality compared to polyclonal Tregs, thereby offering a more precise and effective strategy for preventing allograft rejection.^[^ ^24^, ^25^, ^26^ ^]^ The development of clinically relevant human anti‐HLA‐A2 CAR‐Tregs, capable of effective immune suppression, marked a significant milestone, demonstrating their capacity to protect from graft‐versus‐host disease and human skin transplant rejection in humanized mouse models.^[^ ^27^, ^28^ ^]^ CAR‐Tregs suppressive ability was further enhanced by inducing co‐expression of IL‐10.^[^ ^29^ ^]^ Rapid progress since led to Sangamo Therapeutics initiating the STEADFAST phase I/II trial for HLA‐A2 mismatched kidney transplant patients in 2022 (NCT04817774) and Quell Therapeutics launching a study in 2024 for HLA‐A2 mismatched liver transplant recipients (NCT05234190).

The feasibility of CAR‐Treg therapy critically depends on the efficient and safe delivery of the CAR construct into primary Tregs. However, their less active cell cycle, greater functional sensitivity and limited endocytosis make them notoriously difficult to transfect.^[^ ^30^, ^31^ ^]^ Viral transduction remains the gold standard in CAR‐Treg engineering. However, viral vectors can trigger host immune responses, risk insertional mutagenesis, and have limited packaging capacity (5–15 kbp).^[^ ^32^ ^]^ Their labor‐intensive production and introduction processes significantly contribute to the prohibitive cell manufacturing costs and the resulting limited access to these therapies.^[^ ^33^, ^34^, ^35^ ^]^ Non‐viral physical transfection approaches are promising alternatives thanks to their enhanced immune safety, simplicity, and cost‐effectiveness. Nonetheless the high voltage required for bulk electroporation (BEP) induces Joule heating that significantly alters cell phenotype and decreases viability.^[^ ^36^, ^37^, ^38^, ^39^ ^]^ Lipid‐based transfection and other non‐viral vectors suffer from low efficiency in suspension immune cells due to their reliance on passive and stochastic endocytosis mechanisms.^[^ ^40^ ^]^ Microfluidic cell squeezing has shown excellent performance with immune cell lines such as Jurkat.^[^ ^41^ ^]^ However, their propensity to clogging and the high cost of continuously floating expensive cargo in microfluidic channels has impeded their widespread clinical adoption.^[^ ^42^ ^]^ These limitations underscore the pressing need for advanced transfection techniques that can engineer CAR‐Tregs both safely and efficiently.

Nanoneedles could address the challenge of efficient Treg manufacturing. These arrays of high‐aspect‐ratio nanostructures can access the intracellular environment with minimal disturbance.^[^ ^43^, ^44^, ^45^, ^46^, ^47^ ^]^ Their unique cell interfacing makes them highly effective in delivering a variety of therapeutic agents—including small molecules, nucleic acids, proteins, and nanoparticles—into cells without inducing toxicity.^[^ ^48^, ^49^, ^50^, ^51^, ^52^ ^]^ Moreover, nanoneedles have demonstrated high transfection efficiency across hard‐to‐transfect cell types,^[^ ^53^, ^54^, ^55^, ^56^ ^]^ along with additional advantages such as simpler manufacturing, cost‐effectiveness, and high‐throughput capability.^[^ ^57^, ^58^, ^59^ ^]^ Therefore, nanoneedles hold great potential for developing advanced therapy medicinal products (ATMPs).^[^ ^60^ ^]^ In nanoneedle electroporation (nN‐EP),^[^ ^35^, ^42^, ^61^, ^62^, ^63^, ^64^ ^]^ voltage pulses applied to nanoneedles mediate efficient, localized electroporation, capable of rapidly inducing transient nanopores and efficiently delivering cargo intracellularly.^[^ ^65^, ^66^ ^]^ This method exploits the field enhancement effect at the tip of nanoneedles to achieve electroporation at 10–20 V compared to hundreds of volts required in BEP, minimizing Joule cell damage, and improving safety.^[^ ^67^ ^]^ Despite advancements in nN‐EP,^[^ ^61^, ^67^, ^68^ ^]^ the transfection of primary human immune cells using methods compatible with current good manufacturing practices (cGMP) remains an outstanding challenge for the clinical translation of nanoneedle technology.

Here, we present an efficient and scalable nanoneedle electroporation platform for CAR plasmids transfection of primary human Tregs isolated using cGMP‐compliant protocols. nN‐EP achieved a superior transfection efficiency compared to lentiviral transduction at multiplicity of infection 1 (MOI 1) while retaining cell viability. nN‐EP did not alter the phenotype and proliferation of Tregs, meeting the expansion requirements for clinical applications. The generated CAR‐Tregs exhibited targeted immunomodulatory capabilities, effectively suppressing the proliferation of effector T (Teff) cells in an antigen‐specific manner when co‐cultured with an HLA‐A2^+^ B‐lymphoblastoid cell line (B‐LCL). The results demonstrated that our nN‐EP platform holds significant potential to manufacture CAR‐Tregs efficiently and safely in a cGMP‐compatible manner.

## 2 Results and Discussion

### 2.1 Nanoinjection Yields Inefficient CAR‐Treg Transfection

**Figure 1:**
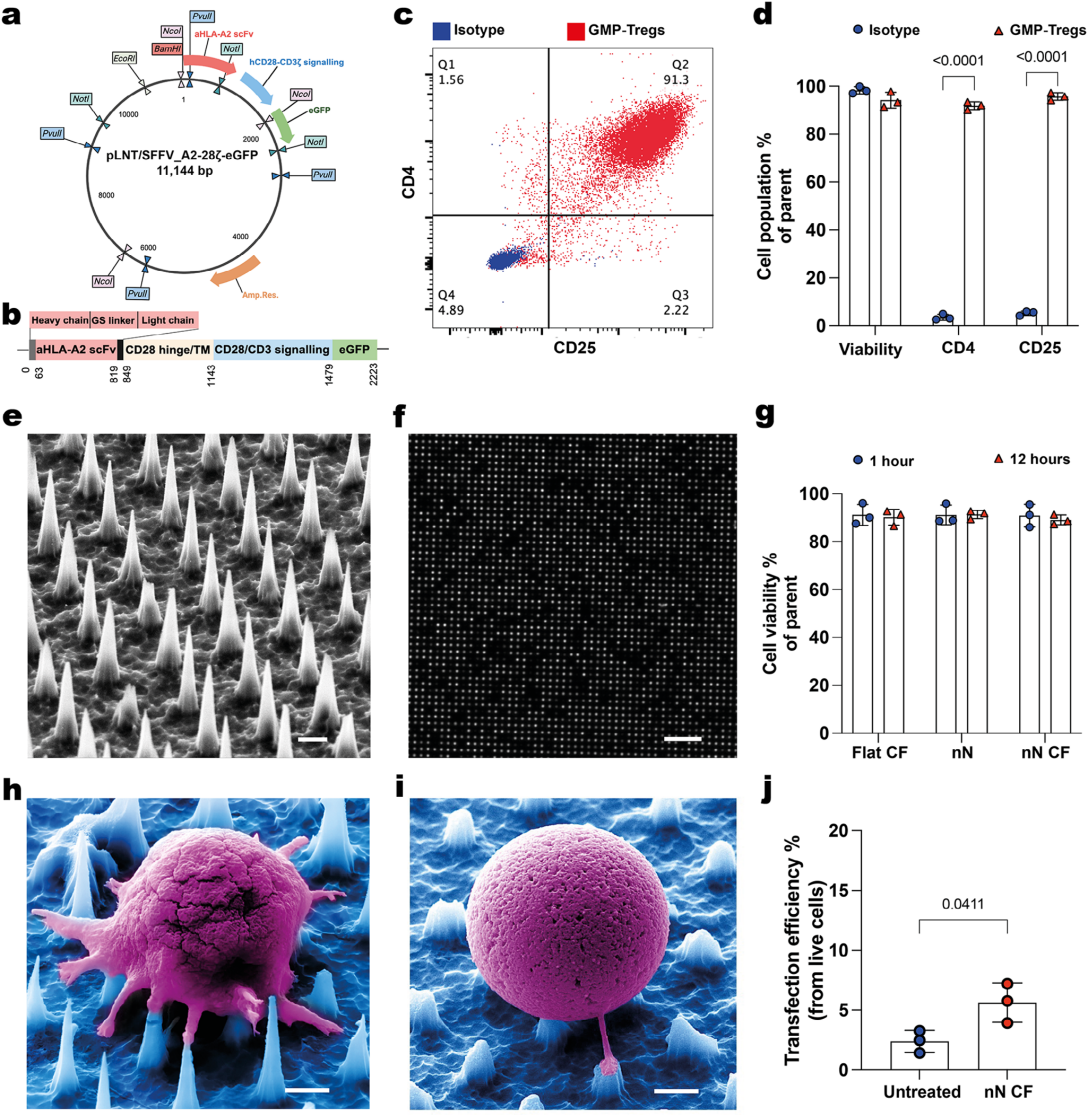
Interfacing Tregs with Nanoneedles. a) Restriction map of the pLNT/SFFV_A2 CAR‐eGFP plasmid used for Treg transfection with nanoneedles. b) Schematic diagram detailing the components of the A2‐28 ζ CAR‐eGFP gene. c) Representative dot plots showing isolated Tregs purity with 91.8% CD4^+^CD25^+^ population in Quadrant 2 (Q2). d) Quantification of live cells, CD4^+^ and CD25^+^ populations obtained from GMP isolation compared to unstained samples (Isotype). Data presented as mean ± standard deviation (SD), *n* = 3, two‐way ANOVA followed by Sidak's multiple comparisons test. *p*‐values are indicated above the bars. (e) SEM image of the nanoneedle arrays. Scale bar: 1 µm. f) Fluorescence image displaying uniform distribution of Cy5‐labeled plasmid across nanoneedles (top view). Scale bar: 10 µm. g) Quantification of Tregs viability after 1 and 12 h on nanoneedles with (nN CF) or without centrifugation (nN) compared to Tregs centrifuged on flat silicon wafers (Flat CF) as control. Data presented as mean ± standard deviation (SD), *n* = 3, two‐way ANOVA. h) False‐coloured SEM image showing interfacial interactions between Treg and nanoneedle surface 1 h post‐interfacing. Scale bar: 1 µm. i) False‐colored SEM image showing recovered spherical shape of Treg and dissolved porous nanoneedle 12 h post‐interfacing. Scale bar: 1 µm. j) Quantification of transfection efficiency by centrifugation‐assisted nanoinjection compared to untreated control. Data presented as mean ± standard deviation (SD), *n* = 3. Unpaired t test. *p*‐value is indicated above the bars.

In this study, we introduced an HLA‐A2‐targeting CAR within primary human regulatory T cells, aimed at promoting immune tolerance in cases of HLA‐mismatched grafts—a central focus in CAR‐Treg therapy for transplantation.^[^ ^24^ ^]^ The second‐generation CAR construct used included an HLA‐A2‐targeting moiety (ectodomain) bound to a CD28‐CD3ζ co‐stimulatory domain and eGFP tracking probe (endodomain) (**Figure** **1**a,b).^[^ ^24^, ^29^ ^]^

We used a cGMP‐compatible protocol to isolate primary human Tregs (GMP‐Tregs), achieving a CD4^+^CD25^+^ cell population purity of > 90% (Figure 1c,d). To transfect Tregs, we initially opted for nanoneedle transfection (nanoinjection) without electroporation, using porous silicon nanoneedles with 3.64 ± 0.14 µm height, 2 µm pitch, 80 ± 10 nm tip width and 600 ± 10 nm base width (Figure 1e). After incubating the nanoneedles with the Cy5‐labeled CAR plasmid, fluorescence imaging confirmed uniform plasmid loading (Figure 1f). We interfaced Treg with nanoneedles either with or without centrifugation at 600 RCF. LIVE/DEAD analysis revealed that nanoneedle interfacing did not affect cell viability, regardless of centrifugation (Figure 1g). SEM imaging at 1 and 12 h post‐centrifugation captured the dynamic of the cell‐nanoneedle interface. At 1 h, Tregs interacted with nanoneedles and extended multiple filopodia, directing them precisely toward the nanoneedle tips to form pivot points upon contact. After 12 h in culture, Tregs recovered their original spherical shape while the nanoneedle dissolved as expected due to their porous silicon composition (Figure 1h,i).^[^ ^43^, ^69^ ^]^ Despite fluorescence microscopy confirming effective plasmid delivery into Tregs, transfection efficiency remained sub‐optimal, staying below 10% in nN CF and comparable to the untreated negative control (Figure 1j).

### 2.2 Nanoneedle Electroporation Yields Efficient Treg Transfection

**Figure 2:**
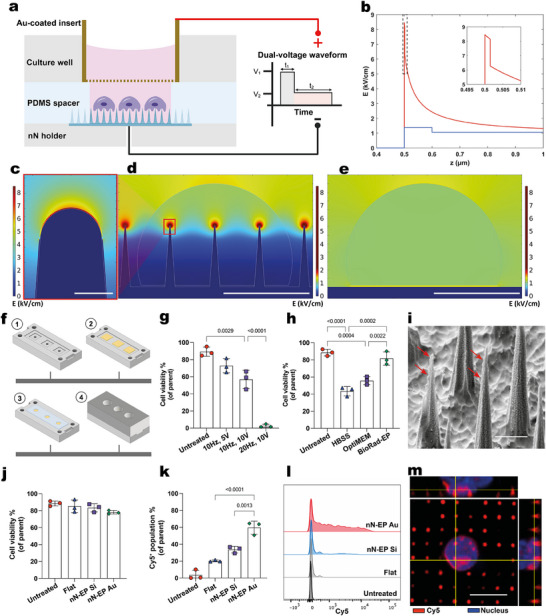
Nanoneedle electroporation of Tregs. a) Schematic illustration of the nN‐EP‐system setup. Not to scale. b) Plot of electric field intensity across a vertical line at the interface starting from the nanoneedle (red trace) or flat electrode (blue trace) side to the cell side. Nanoneedle tip = 8.45 kV cm^−1^. Control planar surface = 1.38 kV cm^−1^. c) Electric field (kV/cm) intensity plot at the nanoneedle tip for the nN‐EP‐system at 10 V, scale bar = 50 nm. d) Electric field (kV/cm) intensity plot for the nN‐EP‐system at 10 V, scale bar = 5 µm. e) Electric field (kV/cm) intensity plot for a flat silicon electrode at 10 V, scale bar = 5 µm. f) Stepwise assembly of the nN‐EP‐system: Stage 1 shows the bottom nanoneedle holder with three square holding areas for holding the nanoneedle chips; Stage 2 shows the nanoneedle chips positioned within the holding areas; Stage 3 shows the PDMS spacer layered over the nanoneedle chips; and Stage 4 shows the fully assembled nanoneedle electroporation well. g) Quantification of cell viability across electroporation conditions. Data presented as mean ± standard deviation (SD), *n* = 3, one‐way ANOVA followed by Tukey's multiple comparison test. *p*‐values are indicated above the bars. h) Quantification of cell viability as a function of electroporation buffer. Data presented as mean ± SD, *n* = 3, one‐way ANOVA followed by Tukey's multiple comparison test. *p*‐values are indicated above the bars. i) SEM image of the Au‐coated nanoneedles. Red arrows indicating gold deposition. Scale bar: 1 µm. j) Viability comparison for cells electroporated under optimized conditions using flat silicon chips (Flat), silicon nanoneedles (nN‐EP Si) and Au‐coated silicon nanoneedles (nN‐EP Au) compared with untreated control (Untreated). Data presented as mean ± SD, *n* = 3, one‐way ANOVA. k) Quantification of Cy5^+^ Tregs population indicative of CAR construct delivery for each electroporation condition. Data presented as mean ± SD, *n* = 3, one‐way ANOVA followed by Tukey's multiple comparison test. *p‐*values are indicated above the bars. l) Representative flow cytometry histogram showing Cy5 fluorescence intensity distribution for each electroporation condition compared to untreated control. m) Representative confocal microscopy image showing Cy5^+^ Treg on nN loaded with Cy5‐tagged plasmid (Red). Treg nucleus was stained with DAPI (Blue). Scale bar: 5 µm.

To improve the low transfection efficiency, we combined nanoneedle interfacing with electroporation (**Figure** **2**a). Finite element simulations of the electric field intensity for a nanoneedle electrode immersed in water, with a flat counter electrode positioned at a distance of 80 µm with an applied voltage of 10 V, revealed a highly localized field enhancement at the nanoneedle tip (8.45 kV cm^−1^) (Figure 2b–d). This enhancement represented a 782‐fold increase compared to the field intensity at the nanoneedle base (0.01 kV cm^−1^) and a six‐fold increase over the field at the cell interface for a planar electrode (1.38 kV cm^−1^) (Figure 2b,e). The field intensity at the nanoneedle tip was higher than the established threshold for electroporation, whereas the base and planar electrode intensities were below the electroporation threshold.^[^ ^70^, ^71^ ^]^ Notably, the peak field amplification was tightly confined to the nanometer‐scale gap between the cell membrane and the nanoneedle tip. These results highlighted the significant electric field amplification achieved by nanoneedle structure, which confined membrane poration to the tips under relatively low voltages. This unique feature of nN‐EP was key to improving both cell viability and transfection efficiency over conventional BEP.

To perform electroporation using nanoneedles, we designed a custom device with two magnetically‐combined sections, SLA 3D printed from biocompatible resins, and an intermediate PDMS membrane for sealing (Figure 2a,e). The lower section positioned the nanoneedles as the base of a cell culture well and provided backside electrical contact through a pogo‐pin electrode, serving as cathode. The upper section formed the well walls, allowing to seed cells over the nanoneedles and to position the frontside electrode at a fixed distance of 80 µm from the nanoneedles. The PDMS membrane, with 6 mm diameter holes was placed between the nanoneedles and the well walls to maintain a watertight seal. After loading the nanoneedles with the A2‐CAR plasmid, the device was assembled, and 3 × 10^5^ Tregs suspended in 200 µL electroporation buffer were added to each well. The device was then centrifuged at 300 RCF for 7 min to facilitate tight interfacing between Tregs and nanoneedles. Following centrifugation, the device was placed in the cell incubator for 15 min, after which the cells were retrieved by pipetting and transferred to a well plate. This workflow resulted in the cell retrieval of 94.22% ± 3.57% of the initially seeded cells (Figure S2, Supporting Information).

We optimized nanoneedle electroporation (nN‐EP) conditions using a dual‐voltage, two train electroporation waveform with a 1 min interval between each train. The waveform consisted of a train of dual‐voltage pulses with frequency f, an initial voltage V_1_ of duration T_1_, a second voltage V_2_ of duration T_2_, and a defined duration T_w_ supplied by an arbitrary waveform function generator (Figure 2a).^[^ ^72^, ^73^ ^]^ First, we determined the optimal V_1_ and f for the nN‐EP waveform^[^ ^73^ ^]^ while keeping the other parameters constant: T_1_ = 100 µs, V_2_ = 5 V, T_2_ = 300 µs, T_w_ = 5 s. Using V_1_ = 10 V with f = 20 Hz in HBSS buffer induced nearly complete cell death, reducing Treg viability to 2.64% ± 1.94%. Lowering the frequency to f = 10 Hz improved viability to 72.9% ± 8.27% for V_1_ = 5 V, and 56.9% ± 10.67% for V_1_ = 10 V (Figure 2f). We chose f = 10 Hz, V_1_ = 10 V since higher voltages increase cargo influx. Using these optimized conditions, we compared electroporation buffers and found that BioRad‐EP maintained the highest cell viability at 81.6% ± 7.73% outperforming optiMEM (55.73% ± 4.7%) and HBSS (43.43% ± 5.45%) (Figure 2g).

We then compared nEP efficiency of natively oxidized 0.01 Ω‐cm, p‐type porous silicon nanoneedles (nN‐EP Si) and gold sputtered ones (nN‐EP Au) (Figure 2h). We anticipated that the naturally oxidized porous silicon surface would primarily act as a capacitive system, with limited electron transfer capability. In contrast, we expected the gold coating to enhance faradaic characteristics, allowing for more efficient electron transfer. Treg viability using the optimized condition V_1_ = 10 V, T_1_ = 100 µs, V_2_ = 5 V, T_2_ = 300 µs, f = 10 Hz, T_w_ = 5 s, and a 1 min interval between the two trains in the BioRad‐EP system, was similar between gold‐coated and native silicon nanoneedles. Viability remained similar to untreated Tregs and those electroporated on flat silicon chips (Figure 2i). However, gold‐coated nanoneedles achieved a significantly higher delivery efficiency at 59.67% ± 7.78% compared to 33.63% ± 3.86% for native silicon and 20.23% ± 1.10% for flat chips (Figure 2j,k). Individual Tregs showed strong Cy5 signals following delivery (Figure 2l).

**Figure 3:**
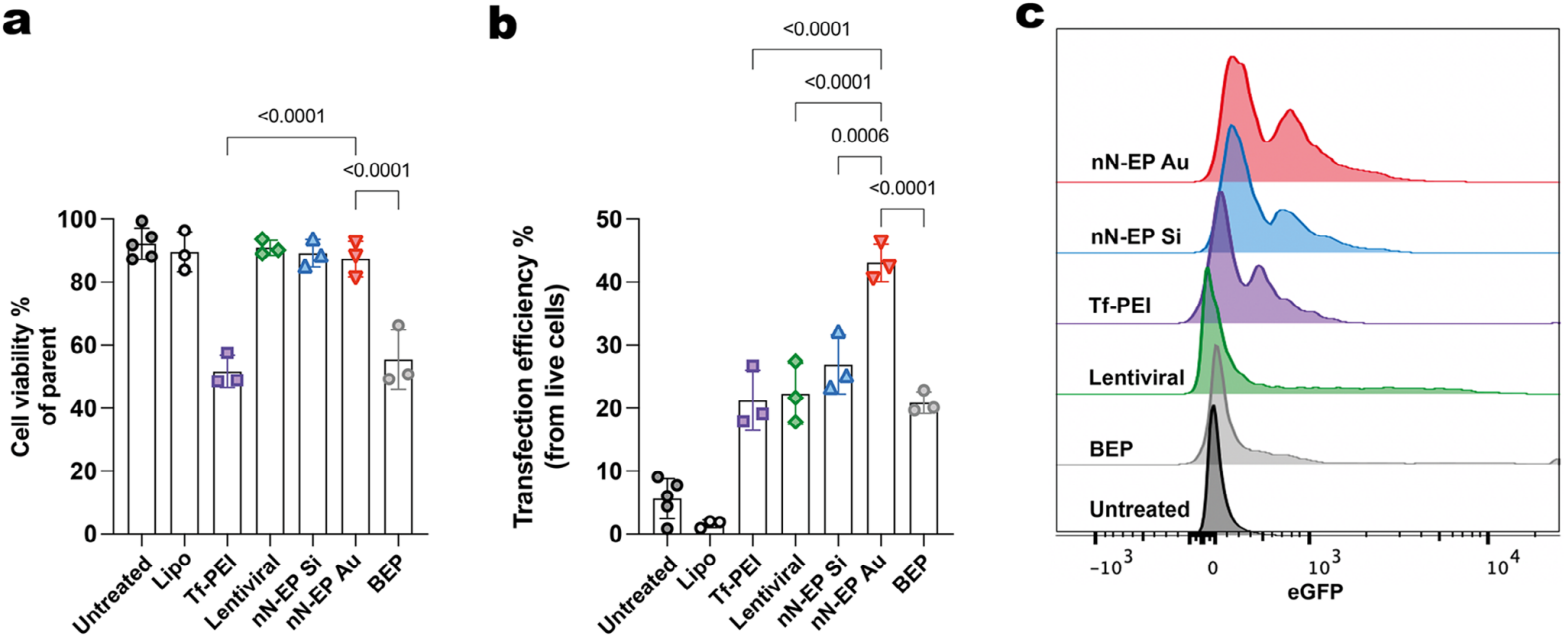
Efficient Tregs transfection via nanoneedle electroporation. a) Quantification of Tregs viability following treatment by lipofection (Lipo), transferrin‐PEI transfection (Tf‐PEI), lentiviral transduction at a multiplicity of infection of 1 (Lentiviral), nN‐EP Si, nN‐EP Au and bulk electroporation (BEP) compared to Untreated Tregs. Data presented as mean ± SD, *n* = 3, one‐way ANOVA followed by Tukey's multiple comparison test. *p‐*values are indicated above the bars. b) Quantification of Treg transfection efficiency for each treatment. Data presented as mean ± SD, *n* = 3, one‐way ANOVA followed by Tukey's multiple comparison test. *p‐*values are indicated above the bars. c) Representative flow cytometry histograms of the distribution of eGFP expression for each treatment.

Next, we compared the transfection efficiency and viability of nN‐EP Si and nN‐EP Au with leading Treg transfection methods, including the gold‐standard lentiviral vector transduction at MOI 1 to guarantee against risks of insertional mutagenesis and phenotypical alterations. Additional methods compared were bulk electroporation (BEP), lipofection (Lipo), and transferrin PEI (Tf‐PEI) (**Figure** **3**). At 2 days following gene transfer, nN‐EP Au achieved the highest transfection efficiency at 43.07% ± 2.96% while maintaining good viability at 87.33% ± 5.65%. The lentiviral vector, carrying the same expression cassette as the CAR‐plasmid, achieved a viability of 90.93% ± 2.39% and a transduction efficiency of 22.28% ± 4.83%. Both Tf‐PEI and BEP groups showed notably lower cell viability (51.63% ± 5.09% and 55.4% ± 9.47%, respectively) and comparable transfection efficiencies (21.33% ± 4.77% and 20.87% ± 1.69%). The Lipo group showed minimal transfection efficiency (1.63% ± 0.6%) and high viability (89.6% ± 6.32%). This data underscored that nN‐EP Au could efficiently transfect primary human Tregs with the A2‐CAR construct (A2‐Tregs) using a cGMP‐compatible protocol while retaining their viability, comparing favorably with the state‐of‐the‐art approach.

Additionally, we evaluated the versatility of the nN‐EP Au for nucleic acid transfection by delivering mRNA. Using the same electroporation parameters as for CAR‐plasmid, nN‐EP Au achieved a transfection efficiency of 31.13% ± 2.80% and a cell viability of 91.33% ± 1.53% (Figure S1, Supporting Information). While these results support the system's adaptability for different nucleic acid cargos, the optimal electroporation parameters for mRNA and plasmid DNA are expected to differ due to their distinct molecular properties. The lower transfection efficiency observed for mRNA likely reflects this mismatch, underscoring the need for further optimization of the electroporation conditions for mRNA delivery in future studies.

### 2.3 Nanoneedle Electroporation Preserves Tregs Phenotype

**Figure 4:**
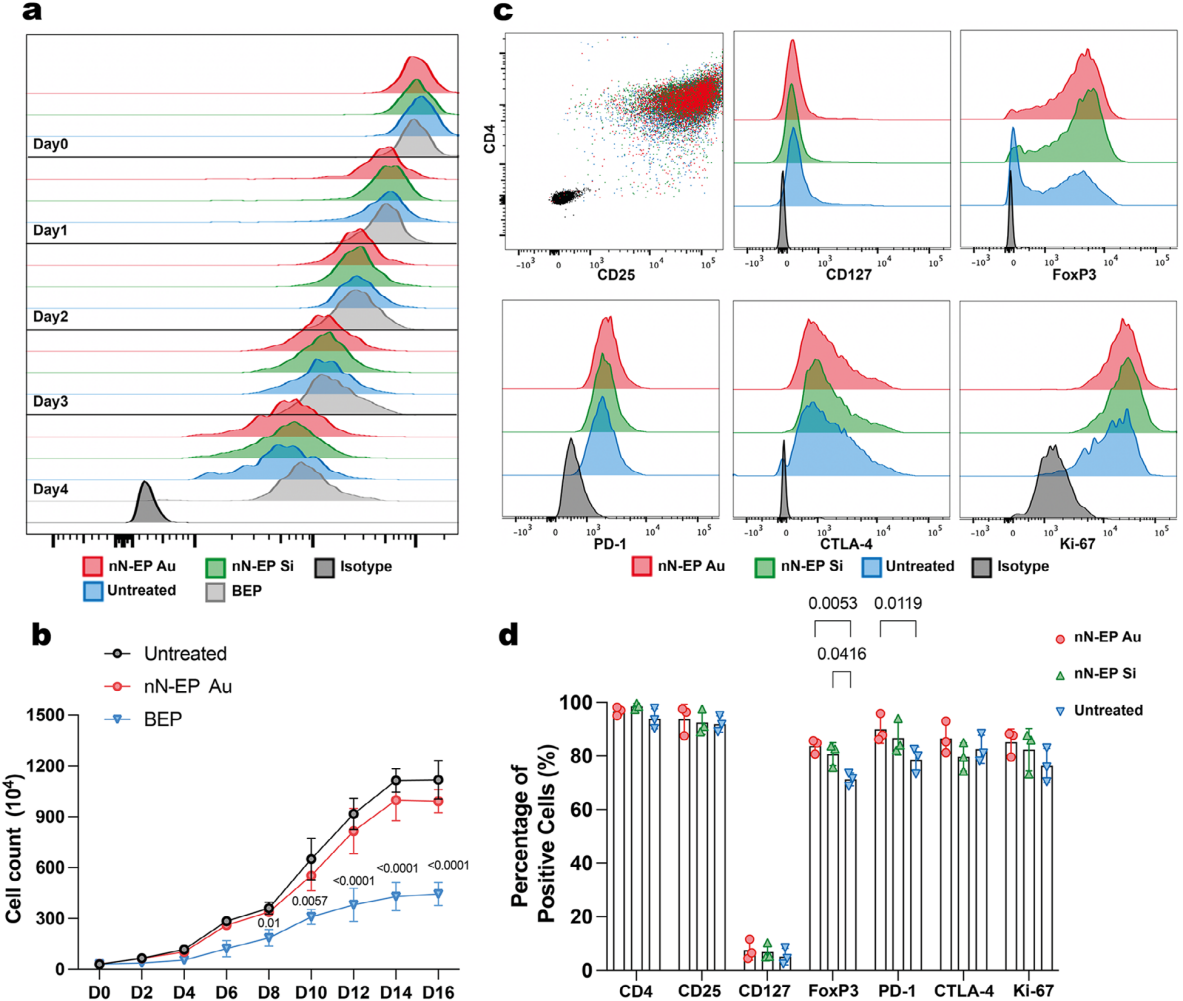
Tregs maintain proliferation and phenotype following nanoneedle electroporation. a) Representative flow cytometry histograms showing CTV fluorescence intensity distribution in Tregs treated by BEP, nN‐EP Si, and nN‐EP Au from Day 0 to Day 4; untreated Tregs served as positive control, and unstained Tregs (Isotype) as negative control. b) Growth curve for A2‐CAR transfected Tregs. Growth curve of untreated, nN‐EP Au, and BEP Tregs over the course of 16 days. Data presented as mean ± SD, n = 3, two‐way ANOVA followed by Tukey's multiple comparison test. *p*‐values comparing BEP and Untreated groups are indicated above the bars. There were no significant differences between nN‐EP Au and Untreated groups. c) Flow cytometry histograms of characteristic Treg markers (CD4, CD25, CD127, and FoxP3), suppression markers (PD‐1 and CTLA‐4), and the proliferation marker Ki‐67. d) Quantification of the expression levels of the Tregs markers in (c). Data presented as mean ± SD, *n* = 3, two‐way ANOVA followed by Tukey's multiple comparison test. *p‐*values are indicated above the bars.

Expanding CAR‐Tregs after genetic engineering is crucial to achieve the cell numbers and functionality needed for effective clinical applications in targeted immunosuppressive therapies. We used a CellTraceViolet (CTV) proliferation assay to assess the expansion potential of A2‐CAR Tregs following electroporation (**Figure** **4**a). The cell growth curve illustrated the long‐term proliferative capacity of A2‐CAR Tregs from untreated, nN‐EP Au, and BEP‐treated groups and was assessed over two weeks (Figure 4b). As expected, given the initial proliferative stimulus at D0, the proliferation of all Treg groups plateaued at D14. BEP‐treated Tregs exhibited significantly reduced proliferative capacity compared to the untreated control, suggesting that BEP treatment may impair long‐term Treg expansion. Notably, from D8 onwards, the proliferation of BEP‐treated Tregs declined significantly and further reduced for the rest of the stimulation cycle. In contrast, nN‐EP Au‐treated Tregs maintained a proliferation rate comparable to untreated Tregs, indicating that nanoneedle electroporation did not compromise long‐term Treg expansion. Importantly, the proliferation index for both nN‐EP Au and nN‐EP Si closely matched that of untreated cells (Figure S3, Supporting Information), further supporting the biocompatibility of nanoneedle electroporation for Treg manufacturing.

After confirming that the cells retained their ability to proliferate, we assessed whether nanoneedle electroporation impacted Treg function. Tregs exert an immunoregulatory effect characterized by high expression of the IL‐2 receptor subunit alpha (CD25) and transcription factor Forkhead box protein 3 (FoxP3), alongside low expression of the IL‐7 receptor subunit alpha (CD127). However, they can be reprogrammed into inflammatory cells when exposed to pro‐inflammatory microenvironment, losing their original characteristics and suppressive functions.^[^ ^74^ ^]^ A2‐Tregs generated by nN‐EP maintained their characteristic phenotype 2 days after transfection, with high purity (> 90%) of CD4^+^CD25^+^CD127^low^ (Figure 4c,d). FoxP3 is a crucial regulator for Treg lineage stability and immunomodulatory function, plays a key role in preventing exhaustion and enhancing CAR‐Treg efficacy.^[^ ^75^, ^76^ ^]^ A larger fraction of nN‐EP Au A2‐Tregs showed FoxP3 (83.77% ± 2.69%) expression than control Tregs (71.27% ± 2.42%, *p* < 0.01). Programmed cell death protein 1 (PD‐1) is important for the maintenance of the suppressive capacity of Tregs. Treg‐Teff cell interactions are partly modulated by the binding of PD‐1 on Tregs surface and programmed cell death ligand‐1 (PD‐L1) on Teffs.^[^ ^77^ ^]^ A higher fraction of nN‐EP Au A2‐Tregs showed PD‐1 (86.53% ± 5.98%) expression compared to control (78.6% ± 4.75%, *p* < 0.05). The upregulation of FoxP3 and PD‐1 suggests an enhanced suppressive ability in nN‐EP Au A2‐Tregs. Cytotoxic T lymphocyte antigen‐4 (CTLA‐4) is an immune regulatory protein binding to CD80 and CD86 on antigen‐presenting‐cells (APCs), inhibiting T cell activation and downregulating immune responses.^[^ ^78^ ^]^ nN‐EP Au A2‐Tregs retain high levels of CTLA‐4 expression, comparable to control. Ki‐67 expression was comparable to control, indicating preserved proliferative capacity. These findings indicated that nN‐EP preserved the phenotypic characteristic of Tregs.

### 2.4 Nanoneedle Electroporated CAR‐Tregs Display Antigen‐Specific Suppressive Capacity

**Figure 5:**
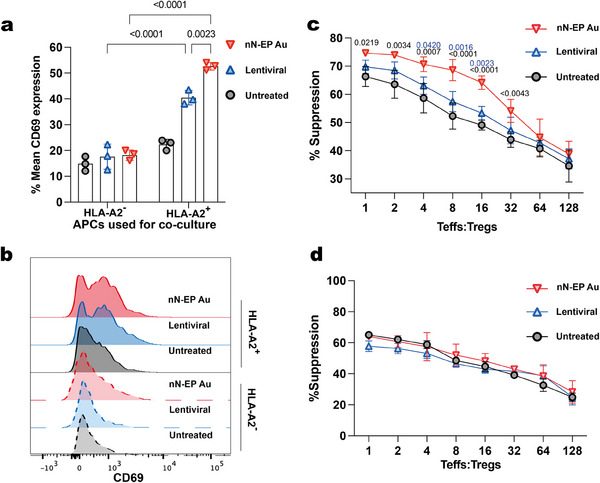
Nanoneedle electroporated Tregs selectively suppress effector T‐Cells (Teffs). a) Quantification of CD69 expression for A2‐Tregs generated from nN‐EP Au and Lentiviral transduction compared to Untreated Tregs after 24 h of co‐culture with HLA‐A2^+^ and HLA‐A2^−^ B‐LCLs. Data presented as mean ± standard deviation (SD), *n* = 3, two‐way ANOVA followed by Tukey's multiple comparison test. *p‐*values are indicated above the bars. b) Flow cytometry histograms showing the distribution of CD69 expression representative of the activation state of Tregs in each group. c) Quantification of the suppressive capacity of untreated Tregs, nN‐EP Au A2‐Tregs and Lentiviral A2‐Tregs when co‐cultured with HLA‐A2^+^ B‐LCLs. Data presented as mean ± standard deviation (SD), *n* = 3, two‐way ANOVA followed by Tukey's multiple comparison test. *p‐*values between nN‐EP Au and Untreated groups (black), and between nN‐EP Au and Lentiviral groups (blue) are indicated above the bars. d) Quantification of the suppressive capacity of untreated Tregs, nN‐EP Au A2‐Tregs and Lentiviral A2‐Tregs when co‐cultured with HLA‐A2^−^ B‐LCLs. Data presented as mean ± standard deviation (SD), *n* = 3, two‐way ANOVA.

Since existing sorting strategies based on FACS to obtain pure A2‐Tregs populations are not yet GMP‐compliant,^[^ ^79^ ^]^ we tested the activation status and suppression ability of the unenriched CAR‐Tregs pools (purity 43.07% ± 2.96%) 2 days after transfection using models of HLA‐mismatch. As expected, incubation with HLA‐A2 positive B‐LCLs APC (A2^+^‐APC) activated both lentiviral and nN‐EP Au A2‐Tregs, increasing CD69 expression (**Figure** **5**a,b). nN‐EP Au A2‐Tregs exhibited the highest CD69 expression level (52.63% ± 1.46%), followed by lentiviral transduction (40.43% ± 2.77%). In contrast, co‐culture with HLA‐A2 negative APC (A2^−^‐APC) resulted in 18.22% ± 1.99% and 17.6% ± 4.86% of A2‐Tregs expressing CD69 in the nN‐EP Au and lentiviral groups, respectively. The CD69 expression of polyclonal Tregs was the lowest (14.87% ± 2.8%) when co‐cultured with A2^−^‐APC and was not significantly upregulated by co‐culturing with A2^+^‐APC. These data supported the desired specific activation of our Tregs in the presence of the A2 antigen.

We further evaluated whether A2‐Tregs displayed the expected antigen‐specific capacity to suppress the proliferation of Teffs (Figure 5c,d). In the presence of A2^+^‐APC, nN‐EP Au A2‐Tregs exhibited superior suppressive function compared to both polyclonal Tregs and lentiviral transduced Tregs. At 1: 1 Tregs: Teffs ratio, Tregs from untreated, lentiviral and nN‐EP Au groups inhibited Teff proliferation by 66.33% ± 3.52%, 69.75% ± 2.4%, and 74.66% ± 0.69%, respectively. At 1: 16 Tregs: Teffs ratio, nN‐EP Au A2‐Tregs exhibited a slower decline in suppression rate (64.15% ± 2.41%), whereas Tregs from untreated and lentiviral groups exhibited 49.09% ± 1.72% and 53.34% ± 2.38%, respectively. Notably, nN‐EP Au outperformed the lentiviral A2‐Tregs likely due to the higher transfection efficiency, yielding a higher enrichment of the A2‐Treg population. When cocultured with A2^−^‐APC, the suppression rate between untreated, lentiviral, and nN‐EP Au groups was comparable at all dilutions. At 1: 1 Tregs: Teffs ratio, the suppression rates were 65.07% ± 2.67%, 57.76% ± 3.43%, and 64.04% ± 0.34%, respectively, lower than that of A2^+^‐APC groups. These findings confirm that the GMP‐compatible nN‐EP manufacturing process generates A2‐Tregs capable of selectively suppressing Teff proliferation in the presence of A2^+^‐APC.

## 3 Conclusion

We showed that centrifugation‐assisted nanoinjection cannot efficiently transfect primary human Tregs. To overcome this challenge, we established a nanoneedle electroporation platform for safe and efficient Tregs transfection. During nanoneedle electroporation, buffer composition, and pulse parameter play a key role determining transfection efficiency and cell viability. Facilitating electron transfer across the liquid‐solid interface by gold coating the nanoneedles increased transfection efficiency, supporting the importance of faradaic electrode behavior in electroporation. This optimization of the electroporation conditions yielded high cell viability (87.33% ± 5.65%), efficient delivery (59.67% ± 7.78%), and transfection efficiency (43.07% ± 2.96%). Importantly, this transfection efficiency surpassed that of viral transduction at MOI 1, the current gold standard for CAR‐Treg manufacturing, and several other established transfection approaches.

The strong electrical field required for bulk electroporation caused high Treg mortality. Surviving cells exhibited reduced proliferation owing to the sublethal damage, a significant disadvantage given the clinical need to expand CAR‐Tregs to tens or hundreds of millions. Additionally, transfection methods using transferrin‐PEI and lipofectamine demonstrated very low efficiency. In contrast, nanoneedle‐electroporated CAR‐Tregs maintained their phenotype and proliferation capacity. Furthermore, they exhibited higher expression levels of suppressive markers such as FoxP3 and PD‐1, potentially contributing to improved immunomodulatory functionality. The nanoneedle‐electroporated Tregs also displayed robust activation in an HLA‐A2 dependent manner, selectively suppressing the proliferation of effector T cells more effectively than transduced Tregs.

Overall, our approach provided a reliable and effective method for manufacturing primary human CAR‐Tregs, compatible with cGMP workflows for isolation, activation, and expansion, capable of meeting regulatory safety and quality standards. Further optimization could involve adjusting pulse parameters, nanoneedle geometry, or surface modifications to improve transfection efficiency. While challenges remain in optimizing transfection efficiency, nanoneedle‐based electroporation already offers a competitive, scalable alternative to viral methods for Treg‐based therapies. Our modular system offers a cost‐effective and scalable approach to manufacturing centimeter‐scale nanoneedle electrodes, enabling large‐scale Treg production. At this scale, individual electrodes can efficiently process the typical cell yield from a patient (10–30 million cells), supporting streamlined and high‐throughput manufacturing. This scalability aims to significantly reduce costs and increase availability to realize the vision of Treg treatment for chronic conditions such as graft‐versus‐host disease, autoimmune disorders and prevention of graft rejection, where repeated administration of CAR‐Tregs may be necessary. The potential to improve Treg manufacturing workflows promises to improve availability of advanced treatments for patients suffering from immune‐related disorders.

## 4 Experimental Section

### Nanoneedle Fabrication

To manufacture the porous silicon nanoneedles according to our established protocol,^[^ ^43^, ^53^ ^]^ a 120–140 nm silicon nitride layer was initially deposited over 0.01 Ω‐cm, boron‐doped p‐type, 100 mm silicon wafers, followed by photo‐lithographically patterned a 2 µm pitch and 600‐nm‐diameter disk array. Before spin‐coating NR9‐250P photoresist, the silicon wafers were dehydrated at 200 °C for 20 min and then pre‐baked at 70 °C for 180 s after spin‐coating. MA/BA6 K‐Suss mask aligner was used for the exposure (exposure configuration: MO HR IFP‐8 holes‐ 365 nm, light intensity: 15.6 mW cm^2^, exposure time: 2.8 s) followed by the post‐baking at 100 °C for 60 s, and development in a 3: 1 (v/v) RD6: de‐ionized water mixture for 12 s. Front‐end reactive ion etching (RIE, Oxford NGP80) was performed in CHF_3_ plasma at 55 mTorr, 150 W, 50 sccm for 155 s, followed by 10 min oxygen plasma treatment (Diener, 100 W, 0.4 mbar). The substrate was then cleaned in a 1: 4 (v/v) mixture of 50% hydrofluoric acid (HF) and de‐ionized water for 120 s, dried through nitrogen steam, then immersed in 0.4 M silver nitrate (AgNO_3_, Sigma–Aldrich) solution (75 mL DI H_2_O, 20 mL 50% HF and 5 mL 0.4 M AgNO_3_) for 120 s. 7 µm in height and 600 nm in diameter nanopillars were fabricated in a mixture of 1% v/v hydrogen peroxide (H_2_O_2_), and HF in DI water solution (316 mL DI H_2_O, 80 mL 50% HF, and 4 mL H_2_O_2_) for 7 min 30 s, then immersing in Type TFA etchant for 10 min to strip the Ag. Back‐end reactive ion etching was performed in SF_6_ plasma at 100 mTorr, 300 W, 20 sccm for 210 s to form the nanoneedles in 3.64 ± 0.14 µm height. The residual silicon nitride layer on the wafer backside was removed by soaking in 50% HF for 30 min. The substrate was diced into chips of 8 mm × 8 mm (DAD3230, DISCO Dicing Saw, Japan).

### Construction of Holders for Nanoneedle‐EP Platform

Nanoneedle‐EP holders and electrodes were designed using Fusion 360 (Autodesk, USA) and printed using a 3D printer (Form 3+, Formlabs, USA) with medical‐grade BioMed Clear Resin (Formlabs, USA). Residual liquid resin on the printed parts was washed off using 99% isopropyl alcohol and the devices were cured at 60 °C for 1 h. After removing the support structures with a handpiece, the devices were disinfected using alcohol‐based disinfectants and UV light. The device consists of two main sections. The bottom section is a rectangular prism with three 8 mm × 8 mm square recesses on its top surface, designed to hold nanoneedle chips. The top section was also a rectangular prism, with its base matching the dimensions of the top surface of the bottom section. This top section has three cylindrical holes that pass through both its top and bottom surfaces. These holes were precisely aligned with the nanoneedle chips in the bottom section, creating wells that hold the cells, with the nanoneedles forming the base of each well. An 80 µm thick PDMS layer, with three holes (6 mm in diameter) created using a biopsy punch, was placed between the two sections to ensure a tight seal and prevent liquid leakage. Each well was designed to hold up to 300 µL of culture media. Electrical contacts were integrated into the device setup, with the anode being provided by a gold‐sputtered tube that fits within the cylindrical holes, featuring a gridded lattice at its base to facilitate even distribution of the electrical field. The cathode connection was established via a pogo‐pin that makes direct contact with the backside of each nanoneedle chip, ensuring efficient electrical conductivity for the electroporation process.

### Finite Element Simulations of Electric Field Distribution Across the nN‐EP Platform

Finite element simulations were performed using the electrostatics physics module in COMSOL Multiphysics 6.2 to analyze the electric field distribution across the nN‐EP platform. A 2D model of the silicon nanoneedle array was created with a height of 3.54 µm, a bottom width of 600 nm, and a tip diameter of 80 nm. Five silicon nanoneedles were evenly spaced 2 µm apart within the computational domain. A cell was placed on top of the platform with a cleft distance of 15 nm at the nanoneedle interface and 100 nm at the flat bottom.^[^ ^80^ ^]^ Symmetry plane boundary conditions were applied to the model edges to approximate an infinitely repeating array, ensuring a representative field distribution while reducing computational complexity. A flat gold electrode, set at 0 V, was positioned 80 µm above the nanoneedles, which were maintained at 10 V. The intermediate medium was modelled as water with a relative permittivity of 80. Cell has a relative permittivity of 103.9.^[^ ^81^ ^]^ An extremely fine physics‐controlled mesh was employed to ensure numerical accuracy, with denser mesh elements near the nanoneedle tips and cell interface to resolve regions of high electric field gradients. The field magnitude plot was generated after the electrostatics study. To visualize the electric field transition across the nanoneedle tips and appreciate the sharp field enhancement, a modified rainbow colourmap was applied, restricting color variation to field intensities between 0 – 2 kV cm^−1^ and assigning the same red color to field intensities above 2 kV cm^−1^.

### Nanoinjection

Porous silicon nanoneedle chips were first treated with oxygen plasma (100 W) for 4 min (ZEPTO‐W6, Diener electronic). They were then immersed in 200 µL 0.1 mg ml^−1^ Poly‐L‐Lysine (25988‐63‐0, Sigma–Aldrich) for 1 h and rinsed with ddH_2_O three times. After that, nanoneedles were incubated with 1 µg µL^−1^ nucleic acid for 30 min and air‐dried.

### SEM Imaging

Cells grown on nanoneedle chips were first rinsed with PBS three times for 5 min each and then fixed with 4% paraformaldehyde (PFA) at 4 °C overnight. Following fixation, substrates were washed three times for 5 min each with chilled milliQ water at room temperature (RT). The substrates were then gradually dehydrated using increasing concentrations of ethanol: 30%, 50%, 70%, 90%, and 96% (each for 10 min), and 100% (twice for 10 min) at RT. Samples were then mounted on SEM stubs and sputter‐coated with a 5 nm layer of gold to enhance their conductivity.

### Nanoneedle Gold Sputter

Gold‐coated nanoneedles were fabricated using direct current (DC) magnetron sputtering (Korvus Technology, UK) in an argon (Ar) atmosphere. The sputtering chamber was evacuated to an initial base pressure of 5×10^−5^ mbar or lower prior to deposition. Argon gas was introduced at a flow rate of 20 sccm to ignite the plasma, which was subsequently stabilized at 10 sccm during deposition. A 7 nm titanium (Ti) adhesion layer was first deposited at a power of 39 W (390 V, 100 mA) with a deposition rate of 0.4 Å/s. This was followed by the deposition of a 35 nm gold (Au) layer at 25.9 W (370 V, 70 mA), with a deposition rate of 1.2 Å/s.

### Human Blood Samples

All human blood samples were obtained from anonymous healthy donors with informed consent and full ethical authorization. Peripheral blood, collected as leukocyte‐enriched blood cones, was supplied by the National Blood Service (NHS Blood and Transplantation, Tooting, London, UK). Ethical approval for this study was granted by the Institutional Review Board of Guy's Hospital under reference number 09/H0707/86.

### HLA Typing of Whole Blood

Blood collected from leukocyte‐enriched blood cones was diluted 1: 1 with sterile PBS. 10 µL of the diluted blood was transferred to a FACS tube and lysed with 200 µL of ACK Lysing Buffer (Thermo Scientific, USA) for 5 min. The cells were then washed twice in PBS at RT. The resulting cell pellets were labeled with 5 µg mL^−1^ anti‐HLA‐A2 antibody PE (BB7.2 clone, Miltenyi Biotec, Germany) for 15 min at 4 °C, followed by washing with PBS. Cells were analyzed by flow cytometry (BD LSR Fortessa, USA), and only HLA‐A2 negative cells were used for antigen specific experiments.

### Isolation of Human Tregs and Teffs

A GMP‐compatible protocol was utilized to isolate CD4^+^CD25^+^ Tregs and CD4^+^CD25^‒^ effector T cells (Teffs) from leukocyte‐enriched blood cones. The isolation process first involved a negative selection step to enrich CD4^+^ T cells, followed by a positive selection to select CD25^+^ cells. Blood from the cones (10 mL cone^−1^) was diluted 1: 1 with sterile PBS and treated with RosetteSep Human CD4^+^ T Cell Enrichment Cocktail (150 µL RosetteSep per 5 mL blood; StemCell Technologies, Canada) for 30 min at RT on the roller. This step crosslinked unwanted CD4^‒^ cells. The blood was further diluted with PBS and layered on top of 15 mL of Lymphoprep per 30 mL diluted blood in a 50 mL Falcon tube. Cells were then centrifuged at 2000 rpm for 20 min, with an acceleration at level 3 and deceleration at level 1. Buffy coat cells were collected and washed with PBS, then resuspended in magnetic‐activated cell sorting (MACS) buffer. CD25 MicroBeads II were added and incubated with cells for 15 min at 4 °C. Cells were washed once to remove excess beads and resuspend in MACS buffer. Cells were added to an LS column placed on a magnetic system (Miltenyi Biotec, Germany). CD4^+^CD25^−^ cells passed through the column and were collected as the Teffs fraction. CD4^+^CD25^+^ Tregs were eluted by removing the column from the magnet, adding 5 mL of MACS buffer, and applying low pressure with a plunger. The purity of Tregs was immediately assessed by flow cytometry using anti‐human CD4 BV605 antibody (SK3 clone, Biolegend, USA) and anti‐human CD25 PE antibody (BC96 clone, Biolegend, USA).

### Tregs Proliferation and Culture

Tregs were cultured at 1 × 10^6^ cells mL^−1^ in X‐VIVO 15 (Lonza, UK) and activated in a polyclonal manner using anti‐CD3/CD28 Dynabeads at 1: 1 bead‐to‐cell ratio (ThermoFisher, UK). The culture media was supplemented with 1000 U mL^−1^ IL‐2 (R&D Systems, Minnesota, USA) and 100 nM rapamycin (LC‐Laboratories, MA, USA).^[^ ^82^ ^]^ The culture medium was refreshed every other day. Day 3 Tregs were utilized in the experimental work.

### Flowcytometric Phenotype Analysis

Cell staining was carried out using 2.5 × 10^5^ cells in 100 µL of PBS. To stain extracellular markers (CD4, SK3 clone, Biolegend; CD25, BC96 clone, Biolegend; CD127, A019D5 clone, Biolegend,) and dead cells, fluorescently conjugated antibodies, and LIVE/DEAD Fixable Near‐IR Dead Cell Stain Reagent (Invitrogen, USA) were added to cell samples according to the manufacturer's instructions. The cells were then incubated at 4 °C for 30 min. After this, to stain intracellular markers, the cells were fixed and permeabilized with the FoxP3/Transcription Factor Fixation/Permeabilization kit (Invitrogen, USA) for 1 h at 4 °C. The cells were then washed with a 1: 10 dilutions of permeabilization buffer in water for 10 min. Following this, cells were resuspended in 100 µL of permeabilization buffer containing intracellular antibodies (FoxP3, 206D clone, Biolegend; Ki‐67, Ki‐67 clone, Biolegend; CTLA‐4, BNI3 clone, Biolegend; PD‐1, EH12.2H7 clone, Biolegend) and incubated for 1 h at 4 °C. After washing, cells were resuspended in 300 µL of PBS and analyzed using the flow cytometer. Compensation^[^ ^83^ ^]^ of each channel was performed using CompBeads (BD, USA).

### ATP Assay

The ATP assay working solution was prepared by combining phenol red‐free medium and ATP‐2D in a 1: 1 ratio. The working solution (100 µL) was added to nanoneedle wells after cell retrieval and to separate cell samples before seeding. For generating a standard curve, specified quantities of cells were seeded into well plates, followed by the addition of 100 µL ATP working solution to each well. The plates were placed on a shaker at 250 rpm for 2 min and then left at room temperature for 10 min to allow the signal to equilibrate. The contents were then transferred to 96‐well black flat‐bottom plate, and luminescence was measured using a Clariostar plate reader.

### Plasmid Production

Certified transformed cells were streaked onto Lysogeny broth (LB)‐agar (Sigma–Aldrich, USA) plates, supplemented with 100 µg mL^−1^ ampicillin (Sigma–Aldrich, USA). Plates were incubated at 37 °C to produce bacterial colonies. An individual colony was then selected using a sterile pipette tip and expanded in 50 mL falcon tubes containing 40 mL of LB medium and 100 µg mL^−1^ ampicillin, shaken at 37 °C for 16 h. Glycerol stocks were prepared by mixing the cell with an equal volume of 50% glycerol (diluted in sterile water). The remaining cell culture was used for minipreparation. Minipreps were performed by centrifuging cell cultures at 6000 RCF for 15 min at 4 °C. DNA was extracted, eluted, and purified from the resulting pellets using EndoFree Plasmid Kit (Qiagen, Germany) following the manufacturer's instructions. DNA concentration was measured using a NanoDrop device, and endotoxin level was assessed using Pierce Chromogenic Endotoxin Quant Kit (Thermo Scientific, USA). The purified plasmid DNA was stored at −20 °C at a concentration of 1 µg µL^−1^.

### CAR‐Plasmid Transfection using Nanoneedle‐EP System

Nanoneedle chips were oxidized by oxygen plasma at 100 W, 0.4 mBar for 4 min prior to use (ZEPTO‐W6, Diener Electronic, Germany). The samples were then placed in chamber slides (ibidi, Germany) and incubated with plasmid (2 µg plasmid/ million cells) at RT for 30 min. 3 × 10^5^ Tregs suspended in 200 µL electroporation buffer were added to each cell culture well in nN‐EP device and centrifuged at 300 RCF for 7 min. The anodes were then inserted into the cell culture wells and connected to an external electricity generator (33522A, Agilent, USA). A dual‐voltage waveform pulse was applied. The optimized conditions were V_1_ = 10 V, T_1_ = 100 µs, V_2_ = 5 V, T_2_ = 300 µs, f = 10 Hz, T_w_ = 5 s, administered twice separated by a 1 min interval. Cells were rested on the nanoneedle device for 15 min and subsequently collected into 24‐well plates by gently pipetting 2 mL of prewarmed Opti‐MEM medium (no phenol red, reduced serum) onto the nanoneedles. After 12 h, the medium was replaced with complete X‐VIVO.

### CAR‐Plasmid Transfection using BEP

Tregs were collected and centrifuged for 5 min at 300 RCF, aspirated, and resuspended in P3 Primary Cell 4D‐Nucleofector X Kit S (V4XP‐3032, Lonza, Swiss) at a concentration of 100 µL per million cells with plasmid (2 µg plasmid/ million cells). Tregs were transferred to Nucleofection cuvette and electroporated using the 4D‐Nucleofector Core Unit (AAF‐1002B, Lonza, Swiss) and X Unit (AAF‐1002X, Lonza, Swiss) with pulse code EH‐115.^[^ ^38^, ^84^ ^]^ Immediately following electroporation, 80 µl medium at RT (X‐VIVO + 5% FBS + 1000 U mL^−1^ IL‐2) was added to the wells of the cuvette strip. Cells were then transferred from the cuvette to 24‐well plates containing pre‐warmed Treg culture media and cultured at 37 °C.^[^ ^85^ ^]^

### CAR‐Plasmid Transfection using Tf‐PEI

The transfection cocktail was prepared using Tf‐PEI Kit (Invitrogen, USA) following the manufacturer's protocol. Tf‐PEI was thoroughly mixed with DNA at an N/P (Nitrogen to DNA phosphate groups) ratio of 6.0. The cell suspension was mixed with Tf‐PEI/DNA complexes (2 µg plasmid/ million cells) and centrifuged at 600 RCF for 1 h at 37 °C, then cultured in cell incubator.

### CAR‐Plasmid Transfection using Lipofectamine

The transfection cocktail was prepared using Lipofectamine 3000 Reagent (Invitrogen, USA) according to the manufacturer's protocol. After a 15 min incubation at RT, the DNA‐lipid complexes were added to cell suspension (2 µg plasmid/ million cells) and centrifuged at 600 RCF for 1 h at 37 °C, then cultured in cell incubator.

### CAR‐Lentivirus Transduction

24‐well non‐tissue culture plates were coated with 50 µg µl^−1^ RetroNectin (Takara, UK) and incubated for 2 h at RT. 50 µL of Tregs prepared at 10 million cells/mL were added to each well. Lentiviral vectors were then added at MOI 1. The volume in each well was topped up to 500 µL with X‐ VIVO 15 complete media (Lonza, UK). Plates were centrifuged at 600 RCF for 1 h at 37 °C and then incubated in an incubator for 3 days.

### Cy5 Tagging of CAR‐Plasmid

To label the CAR construct with a fluorescent Cy5 tag, the Label IT Tracker Intracellular Nucleic Acid Localization Kit (Mirus, Japan) was utilized. A mixture was prepared by combining 5 µg of CAR plasmid with 5 µL of Cy5 label reagent, 5 µL of Labeling Buffer A, and 35 µL of DNase‐free water. The mixture was incubated at 37 °C for 1 h. The Cy5‐labeled CAR plasmid was then purified using ethanol precipitation.

### Tregs Proliferation Assay

Tregs were stained with 5 µM CTV reagent and the fluorescence was recorded as a baseline on Day 0. The stained cells were then transfected according to each experimental group. Immediately following treatment, cells were harvested by gently pipetting and cultured using Tregs culture protocol mentioned above. CTV fluorescence intensity was measured daily from Day 1 to 4 using flow cytometry. Proliferation data was analyzed using FlowJo software.

### B‐LCLs Culture

SPO B‐LCLs (HLA‐A2^+^) and BM21 B‐LCLs (HLA‐A2^−^) were cultured in RPMI‐1640 medium supplemented with 10% heat‐inactivated fetal calf serum, 100 U mL^−1^ penicillin, 100 µg mL^−1^ streptomycin, 2 mM L‐glutamine and 1 mM sodium pyruvate (ThermoFisher, UK).

### Antigen Specific and Non‐Antigen Specific Activation Assay

A2‐Tregs obtained from nN‐EP Au, lentiviral group, and untreated Tregs control were co‐cultured with either HLA‐A2^+^ or HLA‐A2^−^ B‐LCLs at a 4: 1 Tregs to B‐LCL ratio in a 96‐well round‐bottom plate. B‐LCLs were pretreated with 50 µg µl^−1^ mitomycin c (Sigma, USA) at 37 °C for 50 min prior to use. The cultures were incubated for 18 h at 37 °C. Following incubation, the cells were harvested and washed with PBS. To assess cell viability, samples were stained with LIVE/DEAD dye at 4 °C for 30 min. After washing, cells were stained with CD69 PE‐Cy7 (FN50 clone, Biolegend, USA) at 37 °C for 20 min. Cells were washed and resuspended in PBS for analysis.

### Antigen Specific and Non‐Antigen Suppression Assay

A2‐Tregs obtained from nN‐EP Au, lentiviral group, and untreated Tregs control were co‐cultured with either HLA‐A2^+^ or HLA‐A2^−^ B‐LCLs (Treg: B‐LCL ratio = 2: 1), along with autologous HLA‐A2 negative Teffs. The Teffs were labeled with CTV and activated using anti‐CD3/CD28 dynabeads at a 1: 40 bead‐to‐cell ratio. B‐LCLs were pretreated with 50 µg µl^−1^ mitomycin c (Sigma, USA) at 37 °C for 50 min prior to use. After 5 days, CTV fluorescence dilution in Teffs was measured by flow cytometry, and the results were expressed as percent suppression (the inverse of Teffs proliferation) relative to Teffs cultured alone.

### Statistical Analysis

All data were presented as mean ± standard deviation (SD) and were analyzed using GraphPad Prism software (La Jolla, CA, USA). Each data point represents independent measurement, and the error bars indicate the SD. Statistical comparisons were conducted only when at least three independent samples per group were available. The number of independent measurements, statistical methods employed, and significance values (*p*‐values) were detailed in the figure captions.

## Conflict of Interest

The authors declare no conflict of interest.

## Author Contributions

N.J.S. contributed methodology, investigation, formal analysis, project administration, wrote the original draft; C.W. contributed investigation, methodology, and formal analysis; W.E. contributed investigation, methodology, and formal analysis; Y.K.W. contributed investigation, methodology, and formal analysis; C.L.G., S.M. contributed methodology; C.S. provided optimized experimental protocols, performed investigation and validation; D.M. provided optimized experimental protocols; P.Q. provided optimized experimental protocols and formal analysis; X.L. contributed methodology, investigation and formal analysis. P. S. contributed methodology; G.L. contributed supervision, methodology, formal analysis, and project administration; C.C. conceptualized the project, developed methodology, contributed formal analysis, and project administration, acquired resources and funds, wrote the original draft, and supervised the project.

## Supporting information



Supporting Information

## Data Availability

The data that support the findings of this study are available from the corresponding author upon reasonable request.
