# Supplementary material for: Nanoneedle‐Based Electroporation for Efficient Manufacturing of Human Primary Chimeric Antigen Receptor Regulatory T‐Cells
Source: Adv Sci (Weinh). 2025 Apr 15;12(21):2416066. doi: 10.1002/advs.202416066 (PMC12140303; doi:10.1002/advs.202416066)
Supplement: Supplementary file 1 — Supporting Information [file ADVS-12-2416066-s001.docx]

**Supporting Information**

**Nanoneedle-Based Electroporation for Efficient Manufacturing of Human Primary Chimeric Antigen Receptor Regulatory T-Cells**

Ningjia Sun^1^, Cong Wang^1,2,5^, William Edwards^1^, Yikai Wang^1,2^, Chenlei Gu^1,2^, Samuel McLennan^1,2^, Xiangrong L. Lu^1,8^, Panicos Shangaris^3,6,7^, Peng Qi^3^, Daniela Mastronicola^3^, Cristiano Scottà^3,4^, Giovanna Lombardi^3^, Ciro Chiappini^1,2*^

^1^ Centre for Craniofacial and Regenerative Biology, King’s College London, London SE1 9RT, UK

^2^ London Centre for Nanotechnology, King’s College London, London WC2R 2LS, UK

^3^ Peter Gorer Department of Immunobiology, School of Immunology & Microbial Sciences, Faculty of Life Sciences & Medicine, King’s College London, London SE1 7EH, UK

^4^ Department of Life Sciences, Centre for Inflammation Research and Translational Medicine, Brunel University London, UK

^5^ Wenzhou Eye Valley Innovation Center, Eye Hospital, Wenzhou Medical University, China

^6^ School of Life Course & Population Sciences, 10th Floor North Wing, St Thomas’ Hospital, King’s College London, London SE1 7EH, UK

^7^ Harris Birthright Research Centre for Fetal Medicine, King’s College London, London SE1 7EH, UK

^8^ Department of Bioengineering, Imperial College London, SW7 2AZ, UK

E-mail: ciro.chiappini@kcl.ac.uk

**Figure S1. mRNA transfection via nanoneedle electroporation. (a)** Quantification of Tregs viability following nN-EP Au compared to Untreated Tregs. Data presented as mean ± SD, n = 3, Unpaired t test. *p*-value is indicated above the bars. **(b)** Quantification of mRNA transfection following nN-EP Au compared with Untreated control. Data presented as mean ± SD, n = 3, Unpaired t test. *p*-value is indicated above the bars. **(c)** Representative flow cytometry histograms showing eGFP expression levels for nN-EP Au and untreated control.


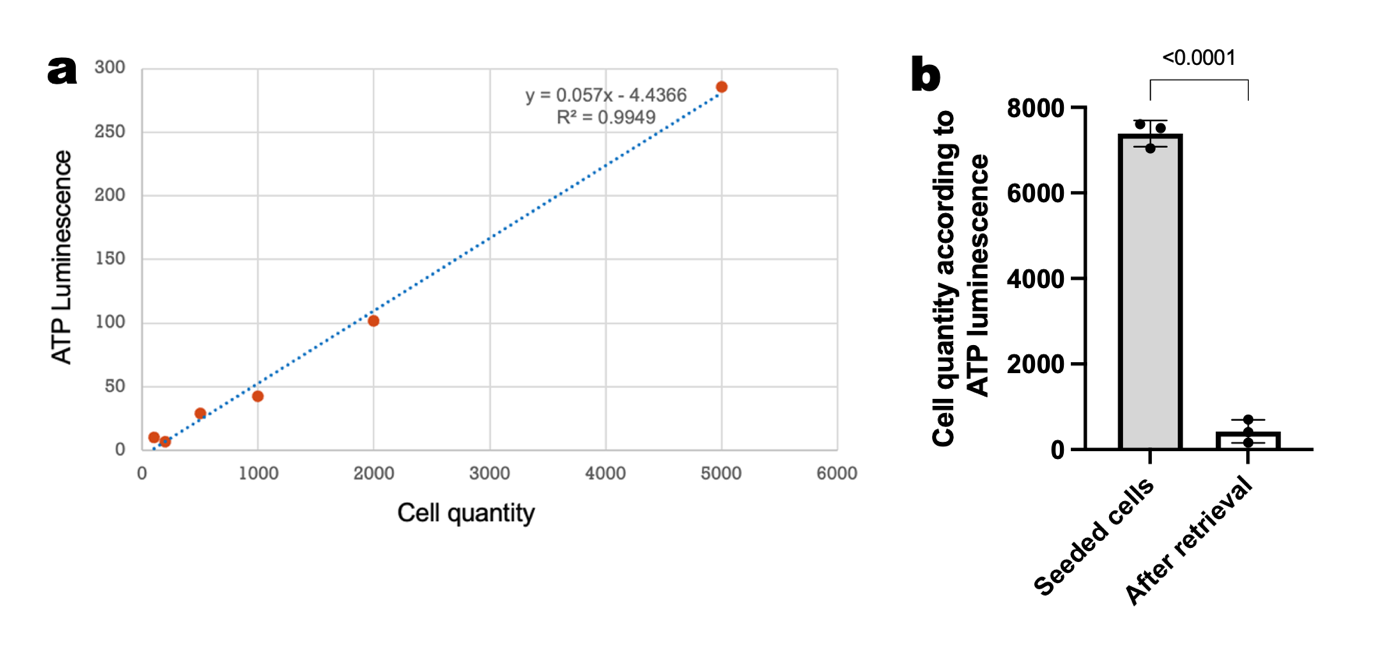


**Figure S2. Retrieval of cells from nanoneedles.** **(a)** Standard curve demonstrating ATP assay linearity in the relevant cell quantification range. **(b)** Bar plot of the number of cells seeded on nanoneedles compared to the number of cells remaining on nanoneedles following retrieval. Data presented as mean ± SD, n = 3, Unpaired t test. *p*-value is indicated above the bars.

**
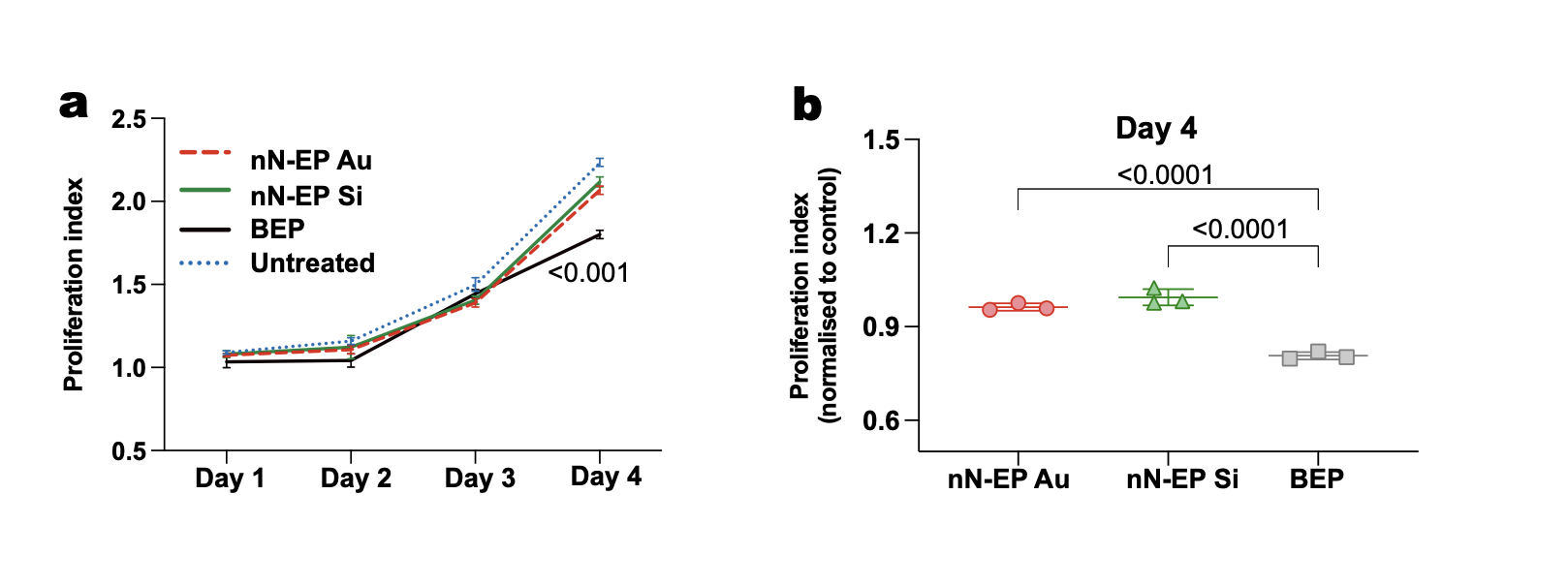
**

**Figure S3. Growth curve for A2-CAR transfected Tregs. (a)** Quantification of the proliferation index showing Treg proliferation from Day 0 to Day 4 for each treatment. Data presented as mean ± SD, *n* = 3, two-way ANOVA followed by Tukey’s multiple comparison test. *p-*value is indicated underneath the bar. **(b)** Day 4 proliferation index normalised to untreated control for each treatment. Data presented as mean ± SD, *n* = 3, one-way ANOVA followed by Tukey’s multiple comparison test. *p-*values are indicated above the bars.
